# The Efficacy of Neuromodulation, Interventional Treatment and Unconventional Therapies in the Treatment of Complex Regional Pain Syndrome: A Systematic Review

**DOI:** 10.7759/cureus.74248

**Published:** 2024-11-22

**Authors:** Essa A Samuel, Khoula Ahmad, Naelijwa J Manongi, Ramkumar Rajapandian, Sajida Moti wala, Esraa M AlEdani, Safeera Khan

**Affiliations:** 1 Physical Medicine and Rehabilitation, California Institute of Behavioral Neurosciences & Psychology, Fairfield, USA; 2 Internal Medicine, California Institute of Behavioral Neurosciences & Psychology, Fairfield, USA; 3 Parkinson School of Health Sciences and Public Health, Loyola University Chicago, Chicago, USA; 4 Trauma and Orthopaedics, California Institute of Behavioral Neurosciences & Psychology, Fairfield, USA; 5 Dermatology, California Institute of Behavioral Neurosciences & Psychology, Fairfield, USA

**Keywords:** chronic pain management, complex regional pain syndrome, interventional pain medicine, neuromodulation, unconventional methods

## Abstract

Complex regional pain syndrome (CRPS) is a chronic debilitating multisystem neuropathic pain disorder. It is characterized by continuous pain, usually out of proportion to any known tissue injury, vasomotor changes, sudomotor or edema, and motor or trophic changes. The objective of this study is to assess the efficacy of neuromodulation, interventional, and unconventional treatments for CRPS. The primary focus is pain reduction, assessed through various scales, with secondary outcomes examining effects on autonomic, sensory, motor, and psychological aspects, and quality of life. PubMed, Cochrane Library, MDPI, and ScienceDirect databases were thoroughly searched using our detailed search strategy and relevant literature compiled. Articles were assessed using our eligibility criteria and quality appraisal tools. All types of study designs were considered. Initially, 463 articles were identified; after a thorough assessment, 23 articles comprising 2307 patients were shortlisted. Neuromodulation interventions, specifically Dorsal Root Ganglion (DRG) and Spinal Cord Stimulation (SCS) demonstrated statistically significant pain reduction (43-82% and up to 70%, respectively). Both modalities demonstrate improvement in secondary outcomes and quality of life. Interventional interventions, specifically nerve blockade ranging from sympathetic, stellate ganglion, and regional nerve blocks, all demonstrate varying levels of efficacy on measured pain and secondary outcomes. Unconventional: Botulinum toxin injections through multiple delivery systems demonstrated varying levels of efficacy in treating pain and improving secondary outcomes. In conclusion, DRG stimulation and SCS, nerve blockade, and botulinum toxin all display promise in alleviating symptoms of CRPS. Definite conclusions were not made due to a lack of clinical trial data, and longer multi-year follow-up is recommended.

## Introduction and background

Complex regional pain syndrome (CRPS) is a chronic debilitating multisystem neuropathic pain disorder; it is characterized by continuous pain usually out of proportion to any known tissue injury, vasomotor changes, sudomotor or edema, and motor or trophic changes [1]. The history of CRPS dates back centuries, with the first recorded description of the condition in the mid-16th century. Since then, the condition has taken on many names, popularly referred to as causalgia, Sudeck's atrophy or dystrophy, reflex sympathetic dystrophy, and even hysteria minor. However, many academics regard the description by American physician Dr. Silas Weir Mitchell, MD, as the birth of what we know today as CRPS, who described burning, unrelenting pain experienced by soldiers during the American Civil War long after bullets had been removed from their wounds, attributing this to nerve injury and the sequelae that followed [2].

The syndrome can be further classified into Complex regional pain syndrome types one and two, which were formerly known as reflex sympathetic dystrophy and causalgia, respectively. They are both purely clinical diagnoses and are currently indistinguishable regarding symptomatology and treatment options. The main differentiating factor is the presence of no apparent nerve injury in type one and known nerve injury in type two. CRPS tends to favor the distal extremities. However, numerous documented cases have involved proximal extremities, spread to contralateral limbs, and cases involving or spreading to the trunk [1]. This syndrome usually manifests in two distinct stages, initially with an acute or inflammatory phase followed by a chronic phase, which is frequently marked by trophic alterations to the soft tissues and even bone [3]. The pathophysiology is poorly understood, with many cases attributed to injury or dysfunction of the small peripheral C-fiber nerves that transmit pain, temperature, and other sensations to the brain. Many activities are also attributed to the development of CRPS symptoms; however, it is also poorly understood why these activities do not lead to the development of symptoms in other patients with the same inciting stimuli. These activities range from fractures, burns, surgeries, sprains and strains, cuts, and rarely penetrating injuries as small as a needle stick [4].

Few studies have been done investigating the epidemiologic factors of CRPS. One population-based study in Olmsted County, Minnesota, found the incidence rate of CRPS one to be 5.46 per 100 000, a period prevalence of 20.57 per 100 000, the median age of onset was 46 with a 4:1 female to male ratio [5]. Another retrospective study of 134 patients referred to a pain clinic in the United States found their mean age to be 37.7 years, with patients experiencing symptoms for 30 months on average before presenting to the pain clinic [6]. Around 70% of the patients were reported as female, while 30% were reported as male. Both studies were conducted in the United States from 1990-1999. A more recent population-based study done in Korea between 2011 and 2015 found their incidence rate to be between 28.0 and 32.0 per 100 000-person years, with CRPS one and two rates being 18.2 and 10.8 per 100 000-person years, respectively. This study also demonstrated a relationship between increasing age and higher CRPS cases, with patients in their 70s having the highest incidence of cases and more than half the patients in the study being over age 50 [7].

The diagnosis of CRPS is largely a clinical one, based on physical examination and symptoms per the Budapest criteria. There is currently no reliable test used solely to diagnose CRPS; however, nerve conduction studies, imaging, and triple-phase bone scans may aid in detecting, localizing disease, and identifying nerve lesions [4]. To meet the Budapest criteria, the patient must have pain out of proportion to the inciting event and at least one symptom from four categories, including sensory, vasomotor, sudomotor/edema, and motor/trophic changes. The patient must also have at least one sign from two of the same four categories during their physical examination, including sensory, vasomotor, sudomotor/edema, and motor/trophic changes [8].

The optimal treatment for CRPS is still unclear, with many treatment options and approaches available. Many experts agree that earlier, more aggressive treatment involving an interdisciplinary team leads to more favorable outcomes and a better prognosis. The more common treatment approaches would include physical and occupational therapies, pharmacology, including drugs such as prednisolone and bisphosphonates, N-methyl-D-aspartate (NMDA) receptor antagonists, opioids, anticonvulsants, and antidepressants, and anti-inflammatory drugs, both non-steroidal and corticosteroids. Interventional treatment options, mainly sympathetic blocks, stellate ganglion blocks, and spinal cord stimulators (SCS), are also commonly employed [1].

In this systematic review, we hope to critically analyze and assess current studies on the efficacy of the treatment options, including neuromodulation and interventional treatment options. Many new and updated studies have been conducted over the last five years, and we hope to provide an updated review of the use of these treatment modalities in treating patients with CRPS. We also hope to critically analyze and assess studies on unconventional therapies used to assess CRPS, including therapies like hyperbaric oxygen, fluid therapy, dry needling, etc. Previous systematic studies and reviews exploring treatment options for CRPS have not included these unconventional treatments in their study design. Given their relatively non-invasive nature and cheap price, we hope to shed light on their usefulness in treating this condition. We hope the information from this study will aid in future guideline-making for clinicians involved in treating CRPS.

## Review

Methodology

This systematic review paper followed the guidelines of the preferred reporting items for systematic reviews and meta-analyses (PRISMA) 2020 [9].

Search Strategies and Data Collection

**Table 1:** Showing the number of articles identified from individual journals MeSH: Medical Subject Heading, MDPI: Multidisciplinary Digital Publishing Institute

Search Strategy	Databases Used	Number of papers Identified
Complex Regional Pain Syndrome and Treatment	Cochrane Library	Five reviews
("Complex Regional Pain Syndromes"[Mesh]) AND ("Complex Regional Pain Syndromes/drug therapy"[Mesh] OR "Complex Regional Pain Syndromes/rehabilitation"[Mesh] OR "Complex Regional Pain Syndromes/therapy"[Mesh]) OR ("Reflex Sympathetic Dystrophy/drug therapy"[Mesh] OR "Reflex Sympathetic Dystrophy/rehabilitation"[Mesh] OR "Reflex Sympathetic Dystrophy/therapy"[Mesh] ) OR ("Causalgia/drug therapy"[Mesh] OR "Causalgia/rehabilitation"[Mesh] OR "Causalgia/therapy"[Mesh])	PubMed (MeSH)	113
Complex Regional Pain Syndrome and Treatment	PubMed	201
Complex Regional Pain Syndrome and Treatment	MDPI	30
Complex Regional Pain Syndrome and Treatment	Science Direct	112

Inclusion and Exclusion Criteria With PICOS Table

Four hundred sixty-three articles were identified and transferred to the endnote, where duplicates were removed. Only articles that were published within the last five years were included. Two researchers then read the title and abstract of each article and applied our inclusion and exclusion criteria. Articles that did not meet the eligibility criteria or were irrelevant to our study were removed. The inclusion and exclusion criteria employed are illustrated in Table 2. The PICOS table describing the study rationale is also illustrated in Table 3.

**Table 2:** Showing inclusion and exclusion criteria for various articles CRPS: Complex Regional Pain Syndrome, ICU: Intensive Care Unit

Inclusion Criteria	Exclusion Criteria
Papers written and published in the English language	Gray literature
Papers focusing on treatment, including neuromodulation, interventional treatment, and unconventional therapies.	Papers including children with CRPS or patients under age 18.
Papers focusing on all adult age groups.	Papers including ICU patients and pregnant women.
Papers written within the last five years.	Papers in which the treatment modality was additive to the main treatment modality being investigated.
Papers including patients with CRPS type one and type two.	Papers in which the treatment modality was investigated were about an animal or organism being tested on human subjects.
Human studies including both males and females.	Papers in which the patients being investigated had multiple etiologies of neuropathic pain, e.g., diabetic neuropathy and differentiation, could not be made as to which patients were suffering from CRPS.

**Table 3:** PICOS table summarizing study rationale PICOS: population, intervention, control, outcome, study design, CRPS: complex regional pain syndrome, ICU: intensive care unit, NRS: numeric rating scale, VAS: visual analog scale, PPR: percentage pain relief, WMD: weighted mean difference

PICOS	Inclusion Criteria	
Population	Patients of all adult ag groups over the age of 18. Patient suffering from CRPS I and CRPS II. Human studies including both males and females.	
Intervention	Any intervention that included interventional techniques, neuromodulation or unconventional therapies.	
Control Group	Any control group or absence of control group.	
Outcome	Change in patient’s pain levels as measured by numeric rating scale (NRS), visual analog Scale (VAS), percentage pain relief (PPR), McGill pain questionnaire and weighted mean difference for pain (WMD). Adverse effects of interventions, persistence of therapeutic effect at follow up, improvement in autonomic, sensory, motor changes, psychological improvement and quality of life improvement.	
Study Design	Case reports and case series, Observational studies including retrospective and prospective, Randomized control trials, Meta-analysis and systematic reviews.	

Risk of Bias Assessment

The quality and risk of bias in the included studies were appraised using several standardized tools. The Cochrane Risk of Bias Tool was applied to randomized clinical trials. For non-randomized clinical trials and observational studies, the Joanna Briggs Institute (JBI) critical appraisal checklists for case reporting and case series were utilized. Additionally, the JBI tool was employed for assessing non-randomized studies.

For systematic reviews, the A Measurement Tool to Assess Systematic Reviews (AMSTAR) checklist was used, while the Scale for the Assessment of Non-Systematic Review Articles (SANRA) checklist was applied to papers lacking a clear methods section.

Two reviewers independently performed a quality appraisal of all remaining articles, ensuring a robust evaluation process. In cases of disagreement, a third researcher was consulted to reach a consensus.

Only studies that met the quality threshold following this comprehensive appraisal process were included in the review. These shortlisted articles were then thoroughly analyzed, and their data were represented in tabulated and graphical formats.

Data Extraction

After the articles met the quality appraisal standards, data extraction focused on identifying key information relevant to the primary and secondary outcomes. The process was conducted by the first and second authors independently, with a third reviewer consulted to resolve discrepancies.

Details extracted included study characteristics, patient demographics, intervention specifics, and outcome measures, as well as any available information on follow-up and study limitations. This structured approach ensured consistency in the data collection process, providing a solid foundation for evaluating the study outcomes.

Statistical Analysis

This study used a qualitative approach to analyze the data. Information from the included studies was carefully reviewed and compared to identify patterns, trends, and differences in the results.

Quantitative outcomes, such as pain relief and adverse effects, were summarized in tables to make it easier to compare the findings. No statistical software or formal calculations were used, as the focus of this review was on describing and comparing the results rather than performing advanced statistical analyses.

Results

Study Selection Process

**Figure 1:**
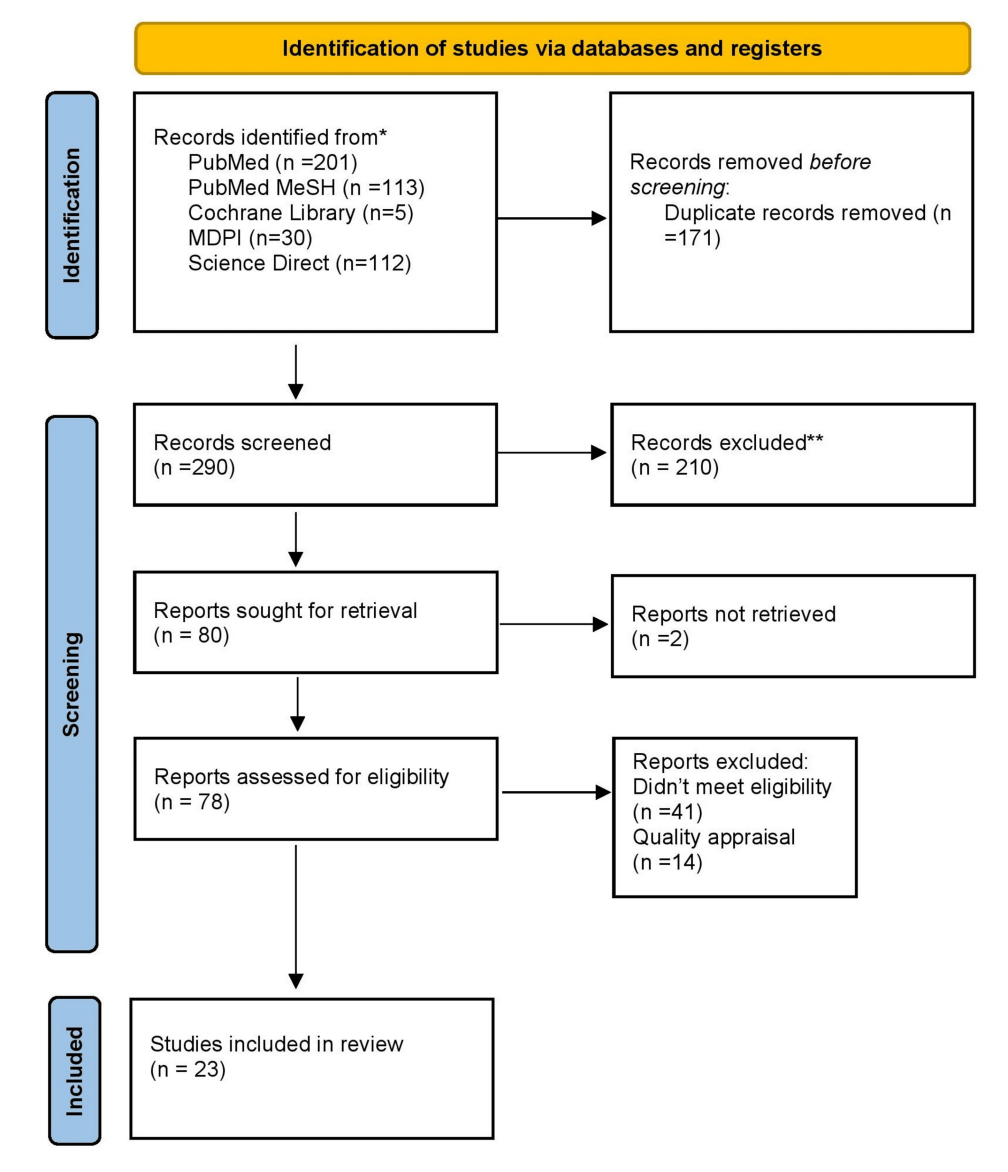
Flow chart showing the process of article selection using the PRISMA guidelines MDPI: Multidisciplinary digital publishing institute, PRISMA: Preferred reporting items for systematic review and meta-analysis, MeSH: Medical subject heading

In total, 461 articles were identified from all the searched journals. After the removal of duplicates, 290 articles remained. Two researchers then thoroughly screened these articles by reading the title and abstract, and 80 studies were identified. The full text was sought for each article; two were excluded since the full text was unavailable. The remaining 78 articles were shortlisted. The shortlisted articles were assessed by applying our eligibility criteria, followed by the quality appraisal process. At the end of applying our eligibility criteria, 37 articles remained, and a further 14 articles were removed after completing the quality appraisal process. In total, the remaining 23 articles were shortlisted for data collection. The study selection process is illustrated in Figure 1 of the PRISMA flowchart [9] (Figure 1).

The articles' suitability was assessed using a variety of quality appraisal instruments, as shown below. Table 4 shows the eligibility of randomized control trials using the Cochrane bias assessment tool; Tables 5-7 show the eligibility of case studies, case series, and observational studies, respectively, using the JBI tool. Table 8 shows the quality appraisal of systematic reviews using the AMSTAR checklist. Table 9 shows the quality appraisal of studies without a clear methods section using the SANRA checklist.

**Table 4:** Showing quality appraisal of randomized control trials using the Cochrane Bias Assessment Tool + indicates yes, - indicates no, ? indicates not clear

Author	Random Sequence Generation	Allocation Concealment	Blinding of participants and personnel	Blinding of outcomes assessment	Incomplete outcome data	Selective reporting	Other bias
Fallico et al., 2021 [10]	+	+	+	?	+	?	+
Mangnus et al., 2023 [11]	+	_	_	_	+	+	+
Halicka et al., 2021 [12]	+	+	+	?	+	+	+
Ratcliffe et al., 2022 [13]	-	?	?	?	+	+	+
Sezgin Özcan et al., 2019 [14]	+	?	-	?	+	+	+
Yoo et al., 2022 [15]	+	+	+	?	_+_	+	+

**Table 5:** Showing quality appraisal of case reports using the Joanna Briggs Institute (JBI) tool Y: yes, N: no

Study Characteristic	Bellon et al., 2019 [16]	Binkley et al., 2019 [17]	Bose et al., 2018 [18]	Caulley et al., 2018 [19]	Chang et al., 2021 [20]	Alkali et al., 2020 [21]	Oh et al., 2021 [22]	Poe et al., 2019 [23]	Roberti et al., 2022 [24]	Rosales et al., 2022 [25]	Sun et al., 2020 [26]	Tereshko et al., 2022 [27]
Were patient’s demographic characteristics clearly described?	Y	Y	Y	Y	Y	Y	Y	Y	Y	Y	Y	Y
Was the patient’s history clearly described and presented as a timeline?	Y	Y	Y	Y	Y	Y	Y	Y	N	Y	Y	Y
Was the current clinical condition of the patient on presentation clearly described?	Y	N	Y	N	Y	Y	Y	Y	Y	Y	Y	Y
Were diagnostic tests or assessment methods and the results clearly described?	N	N	Y	N	N	N	N	N	N	N	Y	N
Was the intervention(s) or treatment procedure(s) clearly described?	Y	Y	Y	N	Y	Y	Y	Y	Y	N	Y	Y
Was the post-intervention clinical condition clearly described?	Y	Y	N	N	Y	Y	N	Y	Y	N	N	Y
Were adverse events (harms) or unanticipated events identified and described?	Y	N	N	N	N	N	Y	N	N	Y	N	N
Does the case report provide takeaway lessons?	Y	Y	Y	N	Y	Y	Y	Y	Y	Y	Y	Y

**Table 6:** Showing quality appraisal of case series using the Joanna Briggs Institute (JBI) tool Y: yes, N: no

Study Characteristic	Gill et al., 2019 [28]	Maihöfner et al., 2018 [29]	Skaribas et al., 2018 [30]	Schwarm et al., 2019 [31]
Were there clear criteria for inclusion in the case series?	Y	Y	Y	Y
Was the condition measured in a standard, reliable way for all participants included in the case series?	Y	N	Y	Y
Were valid methods used for identification of the condition for all participants included in the case series?	Y	Y	Y	Y
Did the case series have consecutive inclusion of participants?	N	N	N	N
Did the case series have complete inclusion of participants?	N	N	N	N
Was there clear reporting of the demographics of the participants in the study?	N	Y	Y	Y
Was there clear reporting of clinical information of the participants?	Y	Y	Y	Y
Were the outcomes or follow up results of cases clearly reported?	Y	N	Y	Y
Was there clear reporting of the presenting site(s)/clinic(s) demographic information?	N	N	N	Y
Was statistical analysis appropriate?	Y	Unclear	Y	Y

**Table 7:** Showing quality appraisal of observational studies using the Joanna Briggs Institute (JBI) tool N/A: Not applicable, Y: yes, N: no

Study Characteristic	Buwembo et al., 2020 [32]	Cheng et al., 2019 [33]	Chmiela et al., 2020 [34]	Delon et al., 2023 [35]	Huygen et al., 2018 [36]	Knife et al., 2020 [37]	Aleanakian et al., 2020 [38]	Risson et al., 2018 [39]	Singh et al., 2022 [40]	Sweeney et al., 2022 [41]	Levy et al., 2019 [42]
Were the two groups similar and recruited from the same population?	Y	Y	Y	Y	Y	N/A	Y	Y	N	y	Y
Were the exposures measured similarly to assign people to both exposed and unexposed groups?	Y	Y	N/A	N	N/A	N/A	Y	Y	Y	y	Y
Was the exposure measured in a valid and reliable way?	N/A	Y	Y	N/A	Y	Y	Y	Y	Y	y	Y
Were confounding factors identified?	N	N	Y	N	N	N	Y	N	N	N	Y
Were strategies to deal with confounding factors stated?	N	N	Y	N	N	N	Y	N	N	N	N
Were the groups/participants free of the outcome at the start of the study (or at the moment of exposure)?	Y	Y	Y	Y	Y	Y	Y	Y	Y	Y	Y
Were the outcomes measured in a valid and reliable way?	Y	Y	Y	Y	Y	Y	Y	Y	Y	Y	Y
Was the follow up time reported and sufficient to be long enough for outcomes to occur?	Y	Y	Y	Y	Y	Y	Y	Y	N	N	Y
Was follow up complete, and if not, were the reasons to loss to follow up described and explored?	Y	Y	Y	N	Y	Y	Y	Y	Y	N	Y
Were strategies to address incomplete follow up utilized?	N	N	N	N	N	Y	N	N	N	N	N

**Table 8:** Showing the quality appraisal of systematic reviews using the Amstar checklist Y: yes, N: no

Study Characteristic	Su et al., 2022 [43]	Wei et al., 2019 [44]	Żyluk et al., [45]
Was an 'a priori' design provided?	Y	Y	N
Was there duplicate study selection and data extraction?	Y	Y	N
Was a comprehensive literature search performed?	Y	Y	Y
Was the status of publication (i.e. grey literature) used as an inclusion criterion?	Y	Y	Y
Was a list of studies (included and excluded) provided?	N	Y	N
Were the characteristics of the included studies provided?	Y	Y	N
Was the scientific quality of the included studies assessed and documented?	Y	Y	Y
Was the scientific quality of the included studies used appropriately in formulating conclusions?	Y	Y	Y
Were the methods used to combine the findings of studies appropriate?	Y	Y	Y
Was the likelihood of publication bias assessed?	Y	Y	Y
Was the conflict of interest included?	Y	Y	Y

**Table 9:** Showing the quality appraisal of studies without a clear methods section using the SANRA checklist

Study Characteristic	Ghaly et al., 2023 [46]
Justification of the article’s importance	1
Statement of concrete aims or formulations of questions	1
Description of the literature search	0
Referencing	2
Scientific reasoning	2
Appropriate presentation of data	2

Study Characteristics

After the critical appraisal process, 23 articles were shortlisted for data extraction and synthesis. These studies comprised 2307 patients, including three randomized clinical studies, 10 case reports and case series, eight observational studies, and two meta-analyses and systematic reviews. These studies were further subdivided into three categories: neuromodulation with 12 studies, interventional treatment with four studies, and unconventional treatment with seven studies. Of the studies excluded, three were randomized clinical trials: Mangnus et al., 2023; Ratcliffe et al., 2022; and Sezgin Özcan et al., 2019 [11,13,14]. Six excluded studies were case reports and case series: Binkley et al. 2019; Caulley et al. 2018; Roberti et al. 2022; Rosales et al. 2022; Gill et al. 2019; and Maihöfner et al. 2018 [17,19,24,25,28,29]. Three of the excluded studies were observational studies: Delon et al. 2023, Singh et al. 2022, and Sweeney et al. 2022 [35,40,41]. One systematic review was excluded: Żyluk et al. (2018), and one study with an unclear methods section was excluded: Ghaly et al. (2023) [45,46].

Primary and Secondary outcomes

The primary outcome investigated in this study was the effect of various interventions on pain, usually measured through standardized scales with values ranging from zero through ten, most popularly including the numeric rating scale (NRS) and visual analog scale (VAS). Other pain scores and scales were also considered, including the percentage pain relief (PPR), the McGill pain questionnaire, and the weighted mean difference for pain scores (WMD).

The secondary outcomes measured include the adverse effects of interventions, the persistence of therapeutic effect at follow-up, improvement in any associated autonomic, sensory, and motor changes, and psychological improvement, including quality of life data where applicable.

Discussion

Neuromodulation in Management of CRPS

Neuromodulation is a rapidly growing and evolving area of pain medicine that incorporates many electrical therapeutic modalities done in a non-invasive or minimally invasive manner that aim to modify, stimulate, regulate, or inhibit electrical or chemical activity in various parts of the nervous system, thus alleviating pain and dysfunction [47]. Many studies on various neuromodulation modalities were analyzed in this review, including articles on dorsal root ganglion stimulation, spinal cord stimulation, various peripheral nerve stimulation modalities, and electroacupuncture. One observational study that included 152 participants and investigated the efficacy of dorsal root ganglion stimulation (DRG) and spinal cord stimulation (SCS) showed promising pain relief; both had comparable response rates to therapy. However, DRG stimulation had greater pain relief and longer-lasting effects at one-year follow-up. Dorsal root ganglion stimulation also did not demonstrate therapeutic habituation at follow-up, unlike SCS. The percentage of pain relief rates of DRG stimulation was calculated to be 82.2% when compared to tonic SCS, which was 77% [42]. Numerous other studies with a much smaller sample size corroborated the effectiveness of DRG stimulation; two additional observational studies had similar outcomes. Huygen et al. showed a 46.8% mean decrease in pain in patients suffering from CRPS type one; patients suffering from causalgia had a mean decrease of 43.7% at one-year follow-up [36]. Another study investigated patients with CRPS of the knee using a more specific neuromodulation intervention of L4 DRG stimulation; the results of this study demonstrated a significant tendency towards normalization of pain parameters at a much shorter follow-up period of three months when compared to the two previously identified studies, which both followed up patients at regular intervals up to twelve months [36-37,42].

One additional case series further provided evidence for the efficacy of DRG stimulation, with all five patients demonstrating significant improvement in pain as measured on the numeric rating scale, the average score pre-procedure being eight to ten reducing to zero to three post-procedure, with the therapeutic effect being maintained at six-month follow-up [30].

Although not as potent as DRG stimulation, spinal cord stimulation showed promising results post-implantation, as documented in numerous observational and case reports. One observational study included 33 participants and showed a 65% improvement in the pain disability index and a 70% improvement in reported pain recorded on a visual analog scale [39]. In addition, three studies reported similar results; one reported an 80% improvement in pain on device placement, and a similarly done case report demonstrated greater than 75% pain improvement that increased to 90% at six weeks [18,23]. A third pain study demonstrated improved pain values from baseline to two-year follow-up; however, the results were not statistically significant [31].

Secondary outcomes were inconsistently reported across all studies; however, in this aspect of identified studies, SCS outnumbered DRG stimulation, with numerous studies reporting an improved quality of life that was statistically significant and a reduction in pain disability index (65%) [31,39]. Other disability indices showed a 38% reduction from reported studies on SCS devices post-implantation at follow-up [18]. Although less reported, improved quality of life and functionality post-procedure were also reported in studies investigating DRG stimulation that persisted at six-month follow-up [30]. Additionally, one study reported decreased rates of depression; however, no effect on thermoreception, mechanoreception, or non-nociceptive perceptions was recorded [37].

Apart from spinal cord stimulation and dorsal root ganglion stimulation, other nerve stimulation modalities appear to manage symptoms of complex regional pain syndrome successfully. One large meta-analysis investigating the effects of electroacupuncture on CRPS and encompassing 1040 patients showed a statistically significant reduction in pain (weighted mean difference of pain scores -1.122 95%CI {-1.682 TO -0.562} P=0.000) of pooled visual analog scale and numeric rating scale scores when compared to conventional therapy. Improved dysfunction, activities of daily living, and detumescence effects were also observed [44]. Direct peripheral nerve stimulation, direct sciatic nerve stimulation, and pulsed radiofrequency all indicated improvement in pain up to 46%, with varying reported secondary outcomes ranging from improved quality of life, reduction in chronic opioid usage up to 21%, and resolution of sleep disturbances at three-month follow-up [22,32,34].

Generally, neuromodulation treatment modalities appeared very safe according to reported adverse events from various studies. Many reported an extremely low rate of adverse events or none at all. The most common adverse event is infection after placement of DRG stimulation devices followed by SCS stimulation devices, usually leading to device removal, sometimes with future device reimplantation [31,34,36]. Table 10 summarizes the studies discussing neuromodulation and their associated results and conclusions. Table 11 also illustrates the summary of adverse events, follow-up periods, limitations, and additional comments on studies related to neuromodulation.

**Table 10:** Summary of studies table for studies related to neuromodulation DRG: dorsal root ganglion, DISNES: direct sciatic nerve electrical stimulation, VAS: visual analogue scale, ADLs: activities of daily living, NRS: numeric rating scale, PRF: pulse radiofrequency, SCS: spinal cord stimulation, EA: electroacupuncture, CRPS: complex regional pain syndrome, PNS: peripheral nerve stimulation

Author and Year of Publication	Type of Study	Number of Participants	Mean Age (years)	Intervention Studied	Results	Conclusion
Bose et al. 2018 [18]	Case Report	1	40	Spinal Cord Stimulation	80% improvement in pain on the placement of the device and 38% reduction in disability index. Improvement in myoclonic jerks.	SCS may be effective in treating pain associated with CRPS I and improving myoclonic jerks.
Oh et al. 2021 [22]	Case Report	1	40	Pulsed radiofrequency of saphenous nerve (PRF)	Improvement in pain as per VAS and resolution of sleep disturbances following procedure sustained at three months follow up.	PRF may be an effective treatment for CRPS II; however, further trials are needed.
Poe et al. 2019 [23]	Case Report	1	59	Spinal Cord Stimulation	Greater than 75% improvement in pain that improved to greater than 90% at six weeks.	SCS may be a favorable treatment for both limb and truncal CRPS where other methods have failed. However, further trials are needed.
Skaribas et al.2018 [30]	Case series	5	Range 49-71	Dorsal root ganglion stimulation	Significant improvement in pain as per NRS, improvement in functionality, and quality of life post-procedure that persisted at six months.	DRG stimulation appears to be effective in treating CRPS of the foot with the persistence of therapeutic effect at follow-up.
Schwarm et al. 2019 [31]	Case series	6	Median Age:43	Spinal Cord Stimulation	Median NRS pain values improved from baseline to 2-year follow-up but were not statistically significant. The quality-of-life score improved significantly at the two-year follow-up.	Pain improvement was not significant; however, reduction in pain scores and improvement in quality-of-life scores prove SCS to be a viable option for managing CRPS.
Buwembo et al. 2020 [32]	Observational	16	Range 26-61	Direct Sciatic Nerve Stimulation (DISNES)	Improvement in pain, 46% reduction in VAS, decreased disability index, and improved quality of life at second follow-up.	DISNES is highly effective in treating pain and autonomic dysfunction related to CRPS I. It also improved quality of life and reduced disability.
Chmiela et al. 2020 [34]	Observational (Retrospective Chart Review)	165(CRPS)	42+/- 11	Direct Peripheral Nerve Simulation	Decrease in pain score (VAS) at 12 months and reduction of patients on chronic opioids (41% from 62% at baseline).	PNS is successful in reducing long-standing pain associated with CRPS, reducing opioid consumption, and improving functional outcomes.
Huygen et al. 2018 [36]	Observational (Prospective Cohort)	11 (CRPS T1) 13 (Causalgia) Total:24	52+/- 11.5	Dorsal Root Ganglion Stimulation	CRPS I patients had a mean decrease of 46.8%, whereas causalgia had a 43.7% mean decrease at 12 months follow-up.	Current evidence supports using DRG stimulation for pain reduction and improved quality of life; however, more clinical trials are needed.
Knife et al.2020 [37]	Observational	12	69+/- 9	Unilateral L4 Dorsal Root Ganglion Stimulation	Significant overall normalization of pain sensitivity and all pain parameters were reduced and trended towards normal at three months.	L4 DRG stimulation successfully decreased pain in patients with CRPS of the knee. Treatment also appears to decrease rates of depression. However, it did not affect thermoreception, mechanoreception, and non-nociceptive perception.
Risson et al. 2018 [39]	Observational	33	48.08	Spinal cord Stimulation	The pain disability index improved by 65% after stimulator implantation, whereas main pain, as measured on VAS, improved by 70%.	Treatment of CRPS I with SCS was effective in improving symptoms of pain and reducing disability.
Levy et al. 2019 [42]	Observational	152	52.5	Spinal cord stimulation vs Dorsal Root ganglion Stimulation	Improvement in Percentage Pain relief in both DRG stimulation patients (82.2%) Vs Tonic SCS (77.0%)	DRG stimulation showed better long-term stable pain relief at 12 months, whereas SCS demonstrated therapeutic habituation.
Wei et al.2019 [44]	Meta-Analysis	1040	Not Reported	Electro- Acupuncture (EA)	Statistically significant reduction in pain as per the weighted mean difference of pooled pain scores (VAS and NRS). There was a statistically significant difference between the EA group and conventional therapy on improving dysfunction, improving ADLs, and detumescence effect.	Further studies need to be conducted; however, current pooled evidence suggests that EA may be effective in treating reflex sympathetic dystrophy post-stroke hemiplegia.

**Table 11:** Summary table describing adverse events, follow-up periods, limitations, and additional comments of studies related to neuromodulation DRG: dorsal root ganglion, SCS: spinal cord stimulation, RCT: randomized control trial, ODI: oswerty disability index, MPSS: mainz pain staging system

Author and Year of Publication	Adverse Events	Follow up Period	Limitations	Additional Comments
Bose et al.2018 [18]	None reported	Not clearly stated, pre- and post-procedure and up to 8 months post-procedure.	None stated	No additional comments
Oh et al.2021 [22]	None reported	It is not clearly stated; follow up at three months.	None stated	No additional comments
Poe et al.2019 [23]	None reported	1,2 and 6 weeks	None stated	No additional comments
Skaribas et al.2018 [30]	One case of CSF leak and post-dural puncture headache.	Pre-procedure,1,2,3 and 6 months	None stated	No additional comments
Schwarm et al.2019 [31]	One case of implanted lead infection, one case of dislocated lead that was surgically corrected	Before the implant,6,12 and 24 months.	Retrospective design. Small sample size	MPSS score:3/3 Mainz pain staging system(chronicity)
Buwembo et al.2020 [32]	None reported	Preimplant, post implant, time 1(2.3 months average), time 2(16.5 months average)	Small sample size	ODI-Oswerty Disability Index improved by 40% and 37% at the first and second follow-ups. Quality of life improvement: Short form survey (SF-36), 69% and 80% improvement at first and second follow-up.
Chmiela et al.2020 [34]	One case of infection leading to device removal	Baseline,1,6 and 12 months	Undocumented visits. Chart review data is weaker than an in-person interview	Peripheral nerve stimulator placement: Sciatic nerve (42%), ulnar (14%), median (8%), Radial (8%).
Huygen et al.2018 [36]	One case of implant site infection, six incidents after permanent placement infections, one motor deficit, and one dural puncture.	Baseline,1,3,6, and 12 months.	Small sample size in each grouping	No additional comments
Knife et al. 2020 [37]	None reported	Baseline,3months	Selection bias. Investigators were non-blinded; therefore, data collection bias was not controlled. No placebo control	No additional comments
Risson et al.2018 [39]	None reported	Not clearly stated, over several years, in most cases	Small sample size	No additional comments
Levy et al.2019 [42]	None reported	1,3,6,9 and 12 months	Underpowered study. The study derived from data from another RCT and was not the primary intention of the study. Expectation bias, Accurate RCT (parent study) was unblinded. Unclear effect of habituation after 12 months. Newer SCS techniques not assessed.	At the end of the trial, the responder rate was 89% DRG, and 86% SCS had greater than 50% pain relief from baseline.
Wei et al.2019 [44]	It could not be confirmed	2 to 6 weeks	Only one included article was English, the remainder being Chinese The difference in technique of administering treatment.	No additional comments

Interventional and Unconventional Treatment in the Management of CRPS

Interventional treatment modalities aim to deliver a therapeutic substance as close as feasible to the anatomic spot in the nervous system responsible for the pain issue; it is essentially a type of targeted drug delivery system to minimize systemic adverse effects [48]. Interventional techniques have been used to treat many medical conditions ranging from headaches to post-herpetic neuralgia and symptoms of peripheral and diabetic neuropathy [49]. Some interventional techniques explored in this article include sympathetic nerve blocks, stellate ganglion blocks, and other regional nerve blocks.

Few studies investigating purely interventional techniques were identified; however, the ones analyzed in this study included two randomized control trials and two retrospective observational studies. Sympathetic nerve blocks were investigated by both Cheng et al. and Yoo et al., and both showed significant pain reduction following the procedure [15,33]. In one study, 155 of the 255 patients suffering from CRPS experienced greater than 50% pain reduction. Of these 155 patients, 132 (85%) experienced this pain relief for approximately one to four weeks or longer [33]. Another study investigated lumbar sympathetic blocks in 48 patients with CRPS, with the twist of adding botulinum toxin type A when administering their injections; the findings proved that lumbar sympathetic blocks are effective in reducing pain, and botulinum toxin is even more effective at reducing pain when compared to only injecting with a local anesthetic [15]. Another interventional technique that showed promise was that of ultrasound-guided stellate ganglion blockade; in one study that included 105 patients, pain reduction was measured to be approximately two points lower on average, measured on the numeric rating scale, with 47% of patients showing greater than 50% pain reduction and 22% no reduction [38]. The last interventional study analyzed in this review included a randomized controlled trial that included 150 patients; it looked at regional nerve blockade with lidocaine combined with citalopram oral therapy. Results indicated a significant improvement of 73.5% Impairment Level Sum Score (ISS) compared to placebo; this score included pain measurement through the Likert scale and McGill pain questionnaire, amongst other parameters. It should be highlighted that this significant improvement occurred in the group that was treated with both lidocaine regional nerve blockade and citalopram oral therapy; two additional control groups were included in this study, one treated with lidocaine nerve blockade and placebo oral therapy and a third with placebo nerve blockade and placebo oral therapy. However, no fourth study arm was included to investigate citalopram without regional nerve blockade [10].

Botulinum toxin was another treatment strategy identified in both categories of interventional treatment and unconventional treatment that included a variety of studies that investigated its usage through numerous different treatment approaches and techniques ranging from intra-articular, intramuscular, subcutaneous, and sympathetic nerve blocks. All included studies showed reduced pain and improved symptoms following treatment to varying degrees. One systematic review was done on the topic and included multiple different modalities of the application of botulinum toxin. This demonstrated significant pain reduction at the first follow-up, approximately one month after treatment; however, no reduction was observed after the second follow-up at two to three months [43]. Two case reports, one investigating intra-articular botulinum toxin injection type A, demonstrated pain reduction at the lateral and posterior shoulder that persisted at a four-month follow-up with an added range of motion improvement [16]. The second case report investigating subcutaneous botulinum toxin injection demonstrated improved pain from 20-70 days post-treatment as measured on the visual analog scale [27].

One interesting randomized control trial investigated the effects of prism adaptation on CRPS patients. This type of procedural learning forces the motor system to adjust to new visuospatial coordinates imposed by objects such as prisms that move the visual field; the degree and strength of the adaptation, once the prisms are removed, can then be measured [50]. The results of this study did not indicate any reduction in pain or associated symptoms following treatment in contrast to previously reported literature on the subject; no improvement was observed in any investigated secondary outcomes, including psychological, autonomic, sensory, or motor, when the treatment group was compared to sham treatment [12].

Various other case reports were analyzed in this study, which included the use of perampanel 4mg, intrathecal baclofen with morphine, and myofascial trigger point dry needling, all of which showed improvement in pain following treatment that persisted at follow-up. However, no additional study designs that included more patients were included in these treatment modalities; therefore, further deductions were not made from these studies [20-21,26].

Data on secondary outcomes were again inconsistent throughout studies on interventional treatments. As discussed earlier, Fallico et al. described an impairment level sum score improvement at 12 months that included an active range of motion, hand skin temperature gap, grip strength, and hand volume gap [10]. In addition, studies on sympathetic and stellate ganglion blocks also demonstrated temperature increases in the affected limb [15,38]. Botulinum toxin, both delivered intra-articularly and subcutaneously, showed evidence of improved quality of life. However, no changes to skin conditions or autonomic symptoms were observed [16,27].

Studies investigating interventional and unconventional treatments were assessed to be extremely safe, with most studies reporting no adverse effects or self-limiting and temporary symptoms [43]. Additionally, the highest rates of adverse effects were seen with stellate ganglion blocks, which reported hematoma formation (0.5%), hoarseness (3.3%), and dysphagia (3.7%) [38]. Table 12 summarizes the results and conclusions of studies related to interventional and unconventional treatment. Table 13 also illustrates the summary of adverse events, limitations, follow-up periods, and additional comments on studies related to interventional and unconventional treatment of CRPS.

**Table 12:** Summary of studies table for studies related to interventional treatment and unconventional treatment RNB: regional nerve block, LSGB: lumbar sympathetic ganglion block, SNB: sympathetic nerve block, VAS: visual analogue scale, NRS: numeric rating scale, MTPDN: myofascial trigger point dry needling, SGB: stellate ganglion block, CRPS: complex regional pain syndrome, RCT: randomized controlled trial

Author and Year of Publication	Type of Study	Number of Participants	Mean Age (years)	Intervention Studied	Results	Conclusions
Fallico et al.2021 [10]	Randomized Controlled Trial	150	56.8	Lidocaine regional nerve block (RNB) plus citalopram oral therapy	There was a remarkable statistically significant improvement in the group treated with both lidocaine RNB and citalopram. 75% improvement in Impairment level sum score at 12 months.	The study provides evidence for the concurrent use of oral citalopram with lidocaine RNB for effective treatment of CRPS.
Halicka et al.2021 [12]	Randomized Controlled Trial	49	Treatment group=47.35 Sham group=45.31	Prism Adaptation	Neither Prism adaptation nor sham groups were associated with a significant reduction in pain intensity. Also, no reduction in psychological, autonomic, sensory, or motor impairment was reported.	Prism adaptation was not associated with pain reduction in long-standing CRPS when compared to sham. Prism Adaptation was also not associated with improvements in secondary outcomes.
Yoo et al.2022 [15]	Randomized Controlled Trial	48	Not stated	Use of Botulinum toxin type A for LSGB	Significantly reduced pain intensity was observed in the group treated with botulinum toxin type A at one and three months. Higher relative temperature increases were observed in the botulinum toxin group compared to the control group.	In LSGB, botulinum toxin type A was more effective than local anesthetic at reducing pain, increasing temperature at three months, and improving cold intolerance in patients with lower limb CRPS.
Bellon et al.2019 [16]	Case Report	1	36	Intra-articular botulinum toxin type A	There was a significant reduction of pain in the lateral and posterior shoulder but no change in the anterior shoulder at one month. The return of pain was lower than the baseline and persistently improved the range of motion at four months. There was no change to trophic skin conditions and autonomic function at follow-up.	Botulinum toxin type A may be beneficial in managing pain associated with CRPS when paired with other treatment modalities. However, larger RCTs are needed.
Chang et al.2021 [20]	Case Report	1	61	Perampanel 4mg	Complete resolution of pain on the administration of the drug, sustained pain relief at seven days and one-month follow-up.	Perampanel might be useful in treating refractory CRPS; further studies are needed.
Alkali et al.2020 [21]	Case Report	1	20	Intrathecal baclofen with morphine	Improvement in symptoms following infusion of intrathecal baclofen with morphine that persisted at ten months follow-up.	CRPS I, unresponsive to conventional six treatment, may benefit from intrathecal baclofen with morphine.
Sun et al.2020 [26]	Case Report	1	61	Myofascial trigger points dry needling	NRS recorded significant improvement in pain following the procedure, which persisted for one year.	MTPDN could be considered a therapeutic option for patients suffering from early-stage CRPS; however, further trials are needed.
Tereshko et al.2022 [27]	Case Report	1	51	Subcutaneous botulinum toxin type A	Improvement in symptoms ranging from 20-70 days after treatment. Pain measures using VAS.	Botulinum toxin type A may improve patients' symptoms and quality of life; however, larger clinical trials are needed.
Cheng et al. 2019 [33]	Observational (Retrospective Cohort)	255(CRPS)	43+/-15	Sympathetic Nerve Blocks (SNB)	155 of 255 patients experienced pain reduction greater than 50%. 85% of these 155 patients, i.e., 132 patients, experienced greater than 50% pain relief for approximately 1-4 weeks.	There was clinically significant pain reduction in CRPS patients treated with SNBs. The success of SNBS is independent of preprocedural temperature.
Aleanakian et al.2020 [38]	Observational (Retrospective)	105	55.6+/-12.4	Ultrasound-guided stellate ganglion blocks	Spontaneous reduction in pain by 2.0 points on NRS. 47% had greater than 50% reduction, with 22% showing no pain reduction. Patients also showed a mean temperature difference of +0.8 °C.	Ultrasound-guided stellate ganglion blocks are safe and reduce pain in CRPS patients.
Su et al.2022 [43]	Meta-Analysis	176	Range 23.8-51	Botulinum Toxin	Pain reduction was significant at the first follow-up (three weeks to one month), assessed by the VAS weighted mean difference. No significant pain reduction at the second follow-up (two to three months)	Definite conclusions were not drawn due to the small sample size of studies; however, Botulinum toxin may be effective in treating CRPS.

**Table 13:** Summary table describing adverse events, follow-up periods, limitations, and additional comments of studies related to interventional treatment and unconventional treatment RNB: regional nerve block, AROM: active range of motion, NRS: numeric rating scale, CRPS: complex regional pain syndrome, SNB: sympathetic nerve block, NaCl: sodium chloride

Author and Year of Publication	Adverse Events	Follow up Period	Limitations	Additional Comments
Fallico et al., 2021 [10]	None reported	1,6 and 12 months	No fourth study arm investigating citalopram without regional nerve block (RNB).	Three trial groups: RNB+ Citalopram, RNB+ placebo oral therapy, Placebo injections (NaCl)+Placebo oral Impairment level sum score: McGill pain questionnaire, AROM, Hand skin temperature gap, grip strength.
Halicka et al., 2021 [12]	None reported	Four weeks before treatment, immediately before and after treatment, four weeks after, three months, and six months after treatment.	Home-based treatment, therefore, compliance is not guaranteed. Deviations from initial treatment instructions are a possibility.	Two trial Groups: Prism adaptation, Sham
Yoo et al., 2022 [15]	None reported	1 and 3 months	Small scale of study. All patients had severe CRPS with intractable pain. No placebo. Limited three-month follow-up.	2 Trial groups: Botulinum toxin group, Local anesthetic the main outcomes measured were pain intensity and temperature change.
Bellon et al., 2019 [16]	None reported	Baseline,1 and 4 months	None stated	No additional comments
Chang et al., 2021 [20]	None reported	Seven days and one month	None stated	No additional comments
Alkali et al., 2020 [21]	None reported	Ten months	None stated	No additional comments
Sun et al., 2020 [26]	None reported	One year	None stated	NRS improved from 7/10 to 3/10.
Tereshko et al., 2022 [27]	None reported	Every three months	None stated	No additional comments.
Cheng et al., 2019 [33]	None reported	Ranges from 10 months to 8 years	Absence of control group. Nonblinding and missing data 83% were lumbar SNB. Variation in conducting procedure.	No addition comments
Aleanakian et al., 2020 [38]	Hematoma (0.5%) Hoarseness (3.3%) Dysphagia (3.7%)	No, the clearly stated follow-up period was between Jan 2007 and December 2017.	Retrospective design with missing data. No control groups. Other treatments being done may affect the results. Only quantitative measurement of pain.	No additional comments
Su et al., 2022 [43]	All self-limited and temporary	1^st^ follow-up up three weeks to 1 month 2^nd^ follow-up 2 to 3 months	Three articles did not state the type of CRPS. A link could not be established between symptoms and treatment effectiveness. Disease duration is not always stated. Modifications to treatment not reported. Two articles did not report the commercial form of drug used.	No additional comments

Limitations

Several limitations were identified while conducting this review. Most studies had a small sample size; therefore, most studies were underpowered. However, it should be noted that CRPS is a rare condition, and finding suitable candidates would prove difficult. Also, there is a lack of randomized control trial data and systematic review/meta-analysis data, as most studies utilized in conducting this review were observational studies and case reports/case series. Variations in the way different interventions were performed, such as nerve blocks, were also a factor. Some studies did not investigate the latest technologies when conducting their research, e.g., spinal cord stimulator devices; therefore, more updated studies are needed to investigate these newer technologies that may be more effective. In most studies there was also limited follow-up of most patients; therefore, the long-term implications of primary and secondary outcomes could not be established. Many studies also included participants who suffered from extremely severe and refractory CRPS; therefore, the data may be skewed.

## Conclusions

The therapeutic modalities of neuromodulation, interventional treatment, and unconventional treatment approaches all demonstrated varying levels of effectiveness in treating the pain associated with Complex Regional Pain Syndrome and other associated parameters, including autonomic, dermatologic, sensory, motor, and psychological symptoms. In the category of neuromodulation, dorsal root ganglion stimulation and spinal cord stimulation both showed promise as effective treatment modalities. Nerve blockade, including sympathetic nerve blocks, stellate ganglion blocks, and regional nerve blocks combined with citalopram oral therapy, demonstrated statistically significant pain improvement and improvement in secondary outcomes. In the last category of unconventional treatments, using botulinum toxin through varying delivery methods showed the most promise as an effective treatment strategy. Definite conclusions were not drawn from any treatment category due to the lack of randomized clinical trial data and the small sample sizes of included studies. However, given the limited clinical data, this review highlights the aspects of CRPS treatment that have been proven to be efficacious thus far. More extensive randomized clinical trials with longer multiyear follow-up intervals are recommended to come to a more definitive conclusion.
